# Negative Affect and Drinking among Indigenous Youth: Disaggregating Within- and Between-Person Effects

**DOI:** 10.1007/s10802-024-01173-1

**Published:** 2024-02-26

**Authors:** Ashley Reynolds, Katie J. Paige, Craig R. Colder, Christopher J. Mushquash, Dennis C. Wendt, Jacob A. Burack, Roisin M. O’Connor

**Affiliations:** 1 Department of Psychology, Concordia University, Montreal, QC Canada; 2 Department of Psychology, University of Buffalo, Buffalo, NY USA; 3 Department of Educational and Counselling Psychology, McGill University, Montreal, QC Canada; 4 Department of Psychology, Lakehead University, Ontario, Canada; 5 Thunder Bay Regional Health Sciences Centre, Ontario, Canada; 6 Thunder Bay Regional Health Research Institute, Ontario, Canada; 7 Northern Ontario School of Medicine University, Thunder Bay, Ontario, Canada; 8 Dilico Anishinabek Family Care, Fort William First Nation, Thunder Bay, Ontorio, Canada

**Keywords:** Adolescence, Indigenous Youth, Alcohol, Drinking, Depression/Anxiety

## Abstract

Negative affect (depression/anxiety) and alcohol use among Indigenous youth in Canada remain a concern for many communities. Disparate rates of these struggles are understood to be a potential outcome of colonization and subsequent intergenerational trauma experienced by individuals, families, and communities. Using a longitudinal design, we examined change in alcohol use and negative affect, and reciprocal associations, among a group of Indigenous adolescents. Indigenous youth (*N* = 117; 50% male; *M*_*age*_=12.46–16.28; grades 6–10) from a remote First Nation in northern Quebec completed annual self-reported assessments on negative affect (depression/anxiety) and alcohol use. A Latent Curve Model with Structured Residuals (LCM-SR) was used to distinguish between- and within-person associations of negative affect and alcohol use. Growth models did not support change in depression/anxiety, but reports of drinking increased linearly. At the between-person level, girls reported higher initial levels of depression/anxiety and drinking; depression/anxiety were not associated with drinking. At the within-person level, drinking prospectively predicted increases in depression/anxiety but depression/anxiety did not prospectively predict drinking. When Indigenous adolescents reported drinking more alcohol than usual at one wave of assessment, they reported higher levels of negative affect than expected (given their average levels of depression/anxiety) at the following assessment. Our findings suggest that when Indigenous youth present for treatment reporting alcohol use, they should also be screened for negative affect (depression/anxiety). Conversely, if an Indigenous adolescent presents for treatment reporting negative affect, they should also be screened for alcohol use.

In Canada, First Nations, Métis, and Inuit peoples are collectively defined as Indigenous peoples. Approximately 4.9% of the population in Canada report an Indigenous identity. These Indigenous peoples have demonstrated tremendous resilience and thrived, despite hardships and traumas that they have experienced and continue to experience (Burack et al., 2014, 2017; Kirmayer et al., 2011). Through colonization and assimilation policies, such as the creation of the government-mandated Indian Residential School system, Indigenous peoples have faced many deleterious consequences including high dropout rates in the current school system (Louie & Gereluk, 2021), an over-representation in the child and family protection system (Ma, 2021), racialized and sexualized violence toward women and girls (Lavell-Harvard & Brant, 2016; Razack, 2016), high rates of incareceration (Chartrand, 2019), and a shorter life expectancy (Tjepkema et al., 2019). While many Indigenous individuals have thrived despite historical traumas and ongoing colonization (Burack et al., 2014, 2017), mental health inequities, including problems with alcohol use and negative affect continue to be reported. In particular, we focused on the development of two indicators of mental health inequities – negative affect and alcohol use – among Indigenous youth that have also been linked to the histories of colonization and oppression (Thira, 2014).

## Depression/Anxiety and Alcohol Use

Negative affect, such as anxiety and depression, often co-occur with alcohol use during the developmental period of adolescence (Marmostein, 2009). Some research indicates the prevalence of depression and anxiety among Indigenous youth may be similar, lower, or higher when compared with non-Indigenous youth (Andrade et al., 2006; Hop Wo et al., 2020; Scott et al., 2022). In a 2021 survey by Statistics Canada (2021), one in five and almost one in four Indigenous youth reported being diagnosed with a mood disorder or an anxiety disorder. However, the prevalence rates may reflect only those individuals who are help-seeking and therefore were able to get a diagnosis. In an American study of the development of depressive symptoms among Indigenous adolescents between the ages of 9 to 13 years old who were followed for 8 years, Martinez and Armenta (2020) found that 63% of Indigenous adolescents experienced depressive symptoms at some point. Moreover, at least 21% of these adolescents demonstrated consistently elevated levels of depression over time and were more likely to develop an alcohol use disorder. Depression and anxiety are well-established comorbid conditions that affect Indigenous peoples and put them at a greater risk for problems with substances, including alcohol (Kenney & Singh, 2016; Rieckmann et al., 2012; Warne et al., 2017; Warne & Lajimodiere, 2015).

Alcohol use rates and patterns vastly differ across Indigenous communities in North America, with high incidences of abstinence among some (Cunningham et al., 2016; Rieckmann et al., 2012) and earlier onset of drinking among others (Cheadle & Whitbeck, 2011). Of concern is the disparities in the consequences associated with alcohol use including high rates of substance use disorders, health complications related to alcohol use, and alcohol-related deaths (Indian Health Service, 2018; Singh et al., 2017; Substance Abuse and Mental Health Services Administration, 2019).

## Negative Reinforcement and the Dual Failure Model

Two dominant models for understanding the risks that lead youth in general to alcohol use include negative reinforcement motives for drinking and the Dual Failure model. Both theories highlight the role of internalizing and externalizing symptoms. Negative reinforcement motives for drinking occur when individuals use substances as a strategy to dampen unpleasant emotions and psychological suffering (Cooper et al., 1995; Kuntsche et al., 2005), suggesting that internalizing symptoms such as negative affect may precede alcohol use. Brockie et al. (2015) found that Indigenous youth who reported high rates of historical loss were at an increased risk for depressive symptoms, PTSD symptoms, and substance use. Additionally, Stewart et al. (2011) found that depressive symptoms were directly linked to drinking to cope, which in turn was linked to heavy alcohol use among Indigenous youth in Canada. Conversely, the Dual Failure Model (Capaldi, 1991, 1992) would suggest that adolescent drinking occurs in the larger context of externalizing symptoms such as rule breaking and aggressive behaviours, which lead to negative consequences and in turn can lead to internalizing symptoms including depressive symptoms (Colder et al., 2013; Paige et al., 2021). Indeed, externalizing symptoms have been found to precede and overlap with substance use among Indigenous youth (Greenfield et al., 2017; Whitbeck et al., 2014).

## The Current Study

In this paper, we introduce the use of analytic approaches that allow for the examination of reciprocal associations between negative affect and alcohol use over time with a group of Indigenous youth. In addition, we will disaggregate between- and within-person effects (e.g., Martinez & Armenta, 2020). Disaggregating within- and between-person effects is in line with the self-medication theory (Khantzian, 1985, 1997), as it invokes individual differences and the utility of considering both between- and within-person-level change over time. According to self-medication theory (Khantzian, 1985, 1997), individuals who have increased symptoms of negative affect (depression/anxiety) tend to drink more (e.g., between-person association). Additionally, if an individual experiences an increase in symptoms of negative affect (depression/anxiety), relative to their typical level of negative affect at one point in time, they are likely to increase their alcohol use, relative to their typical level of alcohol use, at a subsequent point in time (e.g., within-person association). Moreover, reciprocal associations between two constructs imply the disaggregation of between- and within-person effects, as earlier changes in one construct can influence later changes in the other, and vice versa (Curran et al., 2014). We used a longitudinal design and Latent Curve Model with Structured Residuals (LCM-SR; Curran et al., 2014) to examine prospective reciprocal associations and to distinguish within- and between-person associations among a group of First Nations youth from a northern community.

### Hypotheses

Three hypotheses were proposed. One, on average, high levels of negative affect (depression/anxiety symptoms) were expected to be related to high levels of alcohol use over time (between-person level). Two, in considering the individual level, high levels of negative affect (depression/anxiety symptoms) were expected to prospectively predict high levels of alcohol use over time, accounting for average levels of negative affect and alcohol use (within-person level). Three, in considering the individual level, high levels of alcohol use were expected to prospectively predict high levels of negative affect (depression/anxiety symptoms) over time, accounting for average levels of negative affect and alcohol use (within-person level).

## Method

The education administration of the First Nations community approved the battery of measures that were administered to the participants of thi study. Additionally, a subcommittee in the community reviewed and approved this manuscript. The study was approved by the ethics committees at Concordia University and McGill University.

## Participants

Nearly all of the students in grades 6 to 11 (i.e., the end of secondary education in Quebec) were recruited to participate. While most of the students identified as First Nations, some youth identify as Métis or Inuit. Participation was premised on parental consent and participant assent. The parents or legal guardians were provided the option to inform the school if they did not want their child to participate. Additionally, the students were told that they could withdraw from the study at any time. The data were drawn from multiple cohorts during the academic years of 2011–2018. The final dataset included 110 students (44% male; *M*_*age*_=12.46–16.28; grades 6–10). Retention across grades 7 to 11 was 83.6% (*n* = 93), 77.3% (*n* = 85), 82.7% (*n* = 91), 72.7% (*n* = 80), and 56.4% (*n* = 62), respectively. Given attrition and the small number of participants in grade 11, only data from grades 6 through 10 (W1-W5) were used. A full-information maximum likelihood estimation was used to minimize the impact of missing data. This approach allows for inclusion of all of the participants, even those with some missing data.

### Demographics

The participants were asked to indicate their age, gender (0 = female, 1 = male), and grade at each assessment.

### Youth Self-Report (YSR; Achenbach & Rescorla, 2001)

The YSR is a self-report questionnaire that includes 112 items describing behaviour problems that have occurred in the last year among children aged 11 to 18 years. The YSR has been shown to be correlated with other measures of depression in over 50 different cultural groups (Achenbach & Rescorla, 2007). The YSR has also been used to assess depression symptomatology among Indigenous adolescents in the United States, and in comparison to other measures of depression has been found to have strong predictive validity (Thrane et al., 2004). The anxious/depressed subscale (13-items) was used to assess negative affect (Watson, 2005). The participants indicated the truth of each statement on a three-point scale (0 = *not true* to 2 = *very true or often true*) over the past six months. A sum score was derived as a measure of depression/anxiety.

### Self-Report Delinquency Scale (Elliott, Huizinga, & Menard, 1989)

One item from the self-report delinquency scale was used to measure frequency (0 = *never*; 1 = *1–2 times*; 2 = *3–5 times*; 3 = 6–9 times; 4 = *10–19 times*; 5 = *20–39 times*; 6 = *40 + times*) of alcohol use in the past year.

### Data Analytic Strategy

#### Hypothesized Pathways

**Fig. 1:**
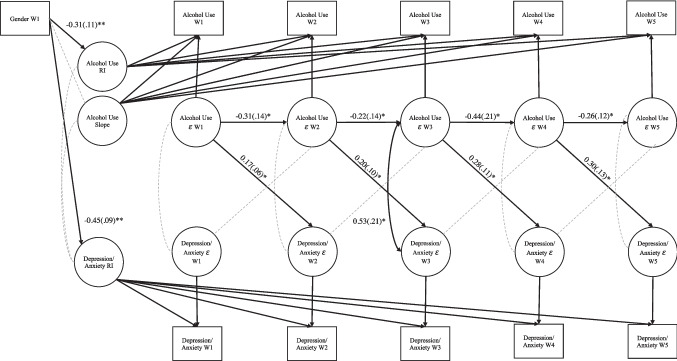
Latent Curve Model with Structured Residuals for Alcohol Use and Depression/Anxiety. *Note*. Solid black lines are significant and dotted grey lines are non-significant pathways. Betas are reported next to hypothesized significant associations and standard errors are reported in parentheses. Levels of significance were based on unstandardized regression estimates. For simplicity, parameter estimates for latent factor loadings are not depicted. Gender was coded such that 0 = female and 1 = male. RI = Random Intercept. W = Wave. ** = p < 0.01. * = p < 0.05

An LCM-SR model (Curran et al., 2014) was used to test the hypotheses because it allowed us to disaggregate within- and between-person effects and test prospective cross-lags (see Fig. 1). A major advantage of the LCM-SR framework is that it imposes a structure onto the time-specific residuals of the observed repeated measures for each construct. Therefore, the residuals are conceptualized as time-specific deviations between the observed repeated measure and the underlying growth curve. This time-specific residual structure represents the within-person portion of the model. The growth factors represent the between-person variance (Curran et al., 2014).

Model building occurred in several steps. First, univariate growth curves for alcohol use and depression/anxiety were tested. Next, we imposed a structure on the time-specific residuals and specified autoregressive and cross-lagged parameters of this residual structure. We then compared the fit of a series of models resulting from imposing equality constraints on several model parameters (i.e., time-specific covariances, autoregressions, and cross-lags). Modification indices and residual correlations were examined, and we considered freeing residual covariances if residual correlations exceeded 0.10 in absolute value (Kline, 2010). Finally, covariates (gender) were added to the model. All of the models were specified in Mplus 8.2 using Full-information Maximum Likelihood estimation (FIML) and Maximum likelihood with robust standard errors (MLR) to account for non-normality in alcohol use (Muthén & Muthén, 1998–2018). Model fit was assessed using conventional absolute and incremental structural equation modeling fit indices. Since cutoffs for “good” fit can vary between models, ranges were used to determine acceptability of model fit (Hu & Bentler, 1999; Marsh et al., 2004). Fit indices and ranges included model chi-square (a significant chi-square indicates poor fit), the comparative fit index (CFI) and Tucker-Lewis index (TLI; for both < 0.90 is poor, 0.90 to 0.94 is acceptable, and ≥ 0.95 is excellent), root mean square error approximation (RMSEA; >0.08 is poor, 0.05 to 0.07 is acceptable, and ≤ 0.05 is excellent), and standardized root mean square residual (SRMR; SRMR, > 0.09 is poor, 0.06 to 0.09 is acceptable, and ≤ 0.06 is excellent). Nested chi-square difference tests were used to assess equality constraints.

#### Power Analysis

Due to the small number of participants, we ran a Monte Carlo simulation to examine power with a focus on the most conceptually important parameters in our proposed model (e.g. regression coefficients and covariances). We chose four sample sizes for descriptive purposes, including the number of participants in the current study (*N* = 110) and others commonly used in psychological science research –: 110, 200, 500, and 1000. The simulation was run using the MONTECARLO command in Mplus 8.2 (Muthén & Muthén, 1998–2018). Parameters from our sample model were used to generate the population covariance matrix and generate sampling distributions of the parameters of interest. We generated up to 500 repetitions and used the common benchmark of 0.80 to indicate adequate power.

## Results

### Descriptive Statistics

Descriptive statistics for the observed variables can be found in Table 1. On average, the adolescents’ alcohol use increased from drinking between 1 and 2 times in the past year at grade six (W1; *M*_*age*_=12.46) to drinking between 3 and 9 times in the past year at grade 10 (W5; *M*_*age*_=16.28). This finding is consistent with evidence that alcohol use typically begins around age 13 years and increases across adolescence among Indigenous youth (Hautala et al., 2019). Consistent with previous empirical evidence that depressive and anxiety symptoms remain relatively stable over the span of adolescence for most youth (Shore et al., 2018; Stapinski et al., 2015; Rice et al., 2019), the participants had a sum score of 6 for self-reported depression/anxiety at grade 6 (W1) and just under a sum score of 5 for self-reported depression/anxiety at grade 10 (W5).

**Table 1:** Bivariate Correlations and Descriptive Statistics

Variable	1	2	3	4	5	6	7	8	9	10
1. Alcohol Use W1	-									
2. Alcohol Use W2	0.38**	-								
3. Alcohol Use W3	**0.55**	**0.48**	-							
4. Alcohol Use W4	0.49**	**0.71**	**0.47**	-						
5. Alcohol Use W5	0.59**	0.36*	0.43**	0.40**	-					
6. Dep/Anx W1	0.30**	0.33**	0.09	**0.51**	0.01	-				
7. Dep/Anx W2	0.27*	**0.41**	0.28*	**0.54**	0.40*	**0.61**	-			
8. Dep/Anx W3	0.16	**0.52**	**0.42**	0.28*	-0.06	**0.54**	**0.57**	-		
9. Dep/Anx W4	0.22	0.17	0.06	0.18	0.24	0.39*	0.47**	**0.64**	-	
10. Dep/Anx W5	0.55**	0.52**	0.36*	**0.51**	0.08	**0.69**	**0.68**	**0.64**	**0.54**	-
**Mean**	0.51	0.76	1.33	1.37	2.24	6.36	5.75	5.56	5.41	4.74
**SD**	1.17	1.30	1.89	1.90	2.09	4.70	5.71	5.31	5.20	4.77
**Skew**	0.58	1.28	1.06	1.07	1.23	2.57	2.01	1.30	1.03	0.47
**Kurtosis**	-0.57	1.22	0.39	0.51	1.02	5.84	3.79	0.46	-0.11	-1.06

*W* wave, *Dep/Anx* depression/anxiety, *SD* standard deviation

* = *p* <0.05, ** = *p <*0.01, Bolded = *p*<0.001

#### Univariate growth models

A linear slope factor was supported for alcohol use (mean slope = 0.42, *p* < 0.001). We added the covariance between the latent random intercept and slope factors, and there was a linear dependency between the factors. Thus, the covariance was not retained. The means and variances of slope factors were nonsignificant across a series of univariate growth curves (e.g., linear, quadratic, piecewise, etc.) for depression/anxiety, indicating no significant growth in depression/anxiety symptoms across our five repeated measures. Accordingly, the subsequent models included a latent random intercept and slope for alcohol use and a latent random intercept, but no slope, for depression/anxiety (see Fig. 1).

### LCM-SR Model

The intercept for depression/anxiety was allowed to covary with the intercept and slope for alcohol use (i.e., the between-person aspects of the model). Regarding the within-person portion of the model, autoregressive paths were supported for alcohol use, but not depression/anxiety. Equality constraints were supported for all cross-lagged paths between the residuals for alcohol use and depression/anxiety as well as autoregressive paths for alcohol use. Finally, two residual correlations were freed for W3 depression/anxiety and W4 depression/anxiety, and W5 alcohol use and W3 depression/anxiety. The final LCM-SR model provided an acceptable fit to the data (χ^2^ (df) = 58.12 (44), *p* = 0.08, CFI = 0.93, TLI = 0.92, RMSEA = 0.04, 90%CI[0.000, 0.064], SRMR = 0.13). Parameter estimates are provided in Fig. 1.

Regarding between-person associations, the variances for the intercepts of alcohol use and depression/anxiety were statistically significant, indicating individual differences in the reporting of initial levels of alcohol use and depression/anxiety. The variance for the slope of alcohol use was also significant, indicating individual differences in the self-reports of change of alcohol use across time. With respect to covariates, gender was significantly related to initial levels of alcohol use and depression/anxiety, as, on average, the females reported higher initial levels of both alcohol use and depression/anxiety. Gender was not associated with changes in alcohol use across time. The covariance between the intercepts was nonsignificant, suggesting that at the between-person level, initial levels of alcohol use were not related to initial levels of depression/anxiety, after accounting for gender. The slope of alcohol use was not significantly associated with the intercept for depression/anxiety, indicating that, on average, the initial levels of depression/anxiety were not related to the growth trajectory of alcohol use.

Within-person associations provided information distinct from the between-person component of the model. The autoregressive paths for alcohol use were significant and negative, indicating that an individual who endorsed higher alcohol use than usual at one wave of assessment also reported lower alcohol use than expected at the following assessment. Within-time covariances between alcohol use and depression/anxiety were nonsignificant across grade 6 (W1) to grade 7 (W2), and grade 9 (W4) to grade 10 (W5). However, the covariance at grade 8 (W3) was significant and positive, indicating that individuals who reported engaging in higher levels of alcohol use than usual also endorsed more depression/anxiety than expected.

With respect to the cross-lags, the prospective associations from depression/anxiety to alcohol use were nonsignificant, suggesting that deviations in depression/anxiety were not related to individual changes in alcohol use at the subsequent assessment. The prospective paths from alcohol use to depression/anxiety were significant and positive. When a participant reported engaging in higher levels of drinking than usual at one timepoint, they also reported more depression/anxiety than expected (accounting for average levels of depression/anxiety) at the following assessment.

### Power Analysis

With 110 participants, a power analysis suggested insufficient power to detect all of the estimated regression coefficients (power ranged from 0.11 to 0.79). The analysis approached adequate power for two regression coefficients, the stability for W3 alcohol use predicting W4 alcohol use (power = 0.79), and W3 alcohol use predicting W4 depression/anxiety (power = 0.70). All remaining power estimates for regression coefficients fell below 0.59. Power was adequate to detect 2 of 7 of the covariances between the within-person residuals (0.05–0.97; we were not powered to detect the smallest coefficients). That is, we were powered to detect the covariance between the within-person residuals for depression/anxiety and alcohol at Wave 3, as well as the covariance between the within-person residuals for depression/anxiety at Wave 3 and depression/anxiety at Wave 4. We were not powered to detect covariance coefficients between the between-person latent slope and intercept factors (0.20–0.75). With 200 participants, power was adequate to detect 3 of 12 regression coefficients (0.15–0.97), 5 of 7 of the covariance coefficients between the within-person residuals (0.05–0.99), and 1 of 2 covariance coefficients between the between-person latent slope and intercept factors (0.34–0.94). At *N* = 500, power was adequate to detect 7 of 12 regression coefficients (0.31–1.00), 6 of 7 of the covariance coefficients between the within-person residuals (0.06–1.00), and 1 of 2 covariance coefficients between the between-person latent slope and intercept factors (0.71–1.00). With regard to power to detect effects at the largest number of participants of 1000, the results were still somewhat variable. Indeed, power was adequate to detect 10 of 12 regression coefficients (0.51–0.97), 6 of 7 of the covariance coefficients between the within-person residuals (0.06–1.00), and both the covariance coefficients between the between-person latent slope and intercept factors (0.95–1.00).

## Discussion

We attempted to disentangle the temporal association of negative affect (depression/anxiety) and alcohol use among Indigenous youth (grades six to grade ten) from a community in northern Canada with a longitudinal design. We used an LCM-SR model to test bidirectional relationships and examine differences in hypothesized associations at between- and within-person levels. Exploring this risk pathway is essential as Indigenous youth mental health challenges are derived from intergenerational trauma and colonization.

With respect to covariates, the finding that, on average, girls reported higher initial levels of negative affect (depression/anxiety) is consistent with the literature on gender differences and adolescent depression/anxiety in both Indigenous and non-Indigenous populations (e.g., Ames et al., 2015; McLaughlin & King, 2015; Salk et al., 2017; Walls et al., 2021). Girls also reported higher initial levels of alcohol use. Epidemiological data suggest that alcohol use has been declining among adolescents and more rapidly for boys, with the exception of drinking alone, which is on the rise and increasing more rapidly for girls (White, 2020). These trends may be leading to a reversal of historical gender differences of males reporting greater alcohol use. In addition, girls may experience internalizing problems, including anxiety and depression earlier than boys (Leve et al., 2005). Future research should be focused on gender differences in negative affect (depression/anxiety) and alcohol use among Indigenous youth.

Our first hypothesis that alcohol use and negative affect (depression/anxiety) would be related at the between-person level was not supported. Initial levels of depression/anxiety and alcohol use were not significantly associated after accounting for gender. This finding is in contrast with evidence of a positive association between depression symptoms and alcohol use among Indigenous peoples, including youth (Schick et al., 2022; Walls et al., 2021). It also differs from Martinez and Armenta’s (2020) finding that when Indigenous youth experienced elevated levels of depression across their adolescence, they were more likely to meet the criteria for Alcohol Use Disorder. We expanded on past work in this area by utilizing a longitudinal LCM-SR model to distinguish between- and within-person associations. Surprisingly, adolescents’ initial levels of depression/anxiety were not significantly related to changes in their alcohol use across time. This contrasts with evidence of an association between depression, anxiety, and later alcohol use among non-Indigenous youth (see Dyer et al., 2019; McCarty et al., 2012, 2013; O’Neil et al., 2011).

Our second hypothesis—that an adolescent’s increased level of negative affect (depression/anxiety) would prospectively predict increases in alcohol use at the within-person level—was also not supported. The findings from the current study are consistent with evidence from non-Indigenous young adults of a unidirectional association between an alcohol use disorder and depression symptoms, but no reverse effect from depression symptoms to an alcohol use disorder (Fergusson et al., 2009). Moreover, we expand on past work by demonstrating that depression/anxiety does not prospectively predict alcohol use at the level of individual change among Indigenous youth. Indeed, when an adolescent reported a higher level of negative affect (depression/anxiety) than was usual for them at one assessment, this deviation was not associated with their change in drinking at the next assessment. This finding is consistent with evidence that negative reinforcement for drinking may not be as relevant during the developmental period of adolescence (e.g., Scalco et al., 2021; Colder et al., 2013). Rather, during the adolescent years, substance use is most likely to occur in a social context (Dishion & Medici Skaggs, 2000; Kobus, 2003; Oetting & Beauvais, 1991). Studies with Indigenous youth have shown that socializing with peers who engage in risky behaviours, including drinking alcohol, were more likely to start drinking at an earlier age (Boyd-Ball et al., 2014) engage in increased monthly alcohol use (HeavyRunner-Rioux & Hollist, 2010) and binge drinking (Chen et al., 2012). When youth experience internalizing symptoms, they may be protected from affiliating with peers who engage in substance use (Fite et al., 2006) and thereby are at a reduced risk for substance use (Colder et al., 2013, 2018; Mason et al., 2008). However, the results from our power analysis suggest that the power to detect the regression coefficients of depression/anxiety predicting alcohol use, especially at the later waves (W3 predicting W4 and W4 predicting W5), on the within-person side of the model was considerably lower than the power to detect the regression coefficients of alcohol use predicting depression/anxiety across all simulated models. Therefore, another possible explanation for our null findings is low power, and future research in this area should aim to utilize considerably larger groups to examine within-person associations between negative affect (depression/anxiety) and alcohol use among Indigenous youth.

Our third hypothesis—that high levels of alcohol use will prospectively predict high levels of negative affect (depression/anxiety) at the within-person level—was supported. When an adolescent endorsed a higher level of drinking than was usual for them at one assessment, they had higher levels of depression/anxiety than expected at the next assessment. This finding is consistent with findings among non-Indigenous youth whereby binge drinking was associated with subsequent depression symptoms one year later (McCabe et al., 2023). However, this is a novel finding among Indigenous youth, suggesting that alcohol use precedes negative affect.

The study of alcohol consumption among Indigenous communities must also involve the consideration of systemic factors (i.e., colonization) that contribute to mental health problems in this population (Gone, 2021). For example, colonization and assimilation policies have directly impacted Indigenous peoples’ cultural practices and identities (Chase, 2012; Sszlemko et al., 2006), which in turn may account for alcohol consumption and related consequences (Brave Heart, 2003; Ross et al., 2015; Whitbeck et al., 2004; Wiechelt et al., 2012). Specifically, drinking alcohol can pull Indigenous youth away from engaging in traditional activities and forming their cultural identities, and thereby lead to negative affect. This implies that the prospective links between alcohol use and negative affect may arise from consequences associated with problematic alcohol use. Conversely, building a sense of pride and belonging to one’s ancestral culture can be protective (Burack et al., 2014, 2017; Brown et al., 2021). For example, adolescents who value cultural activities may be protected from engaging in risky behaviours, including alcohol use, because they offer an alternative reinforcement to drinking (Spillane et al., 2020; Goldstein et al., 2021).

Another novel finding was that when an adolescent endorsed a higher level of drinking than was usual for them at one assessment, their alcohol use was lower than usual for them at the next assessment. As Indigenous youth are more likely to experience negative consequences associated with their drinking (Indian Health Service, 2018; Landen et al., 2014; Stanley et al., 2014), the consequences may deter heavy alcohol use at the following assessment. Additionally, these alcohol-related consequences may also lead other individuals in the community to help the adolescent reduce their alcohol consumption. However, this interpretation warrants further investigation.

## Limitations and Future Directions

This study has some limitations. One, the project involves Indigenous youth from a single First Nation in northern Quebec. We consider this study to be a first step to gathering information from various communities independently, which can help identify the ways in which each respective community show similarities and differences in the developmental patterns of alcohol use and negative affect (depression/anxiety). Although the current findings are not immediately generalizable to other Indigenous youth in Canada or the United States, this approach is consistent with the methodological perspective that the inclusion only of youth from a single community is preferable in that findings from homogenous grouping are more precise and reliable than those from heterogenous groups. Two, only youth who attended school were recruited. While nearly all of the youth in this community attend the school, the relevance to those who do not regularly attend or are not enrolled in school should be examined. Three, the current study was limited by how negative affect was measured. Although depression and anxiety are highly correlated, both theory and research would suggest that they also have independent features. For example, while the items are generally used to assess negative affect and psychological distress, the physiological hyperarousal may be unique to anxiety and the low positive affect may be specific to depression (Anderson & Hope, 2008; Chorpita, 2002; Clark & Watson, 1991), which in turn may have independent pathways to predicting drinking behaviour (e.g., Schleider et al., 2019). Further, the YSR has not been adapted to local Indigenous worldviews and expressions of depression/anxiety. Defining and measuring depression and anxiety in ways that are culturally meaningful to Indigenous youth is key to ensuring construct validity (Beals et al., 2005; Whitbeck et al., 2014). Yet, this work remains limited among Indigenous populations, as the DSM-5 criteria for Major Depressive Disorder and Generalized Anxiety Disorder are based on Western conceptualizations and do not consider Indigenous knowledge and concepts of health and wellness related to depression.

Four, the small number of participants only provided power to detect moderate sized effects; and thus, we may have missed conceptually interesting small effects. Relatedly, we were unable to test moderation or include certain covariates in our model due to limited power. Namely, past findings suggest the importance of considering externalizing symptoms and how it influences risk for substance use among Indigenous youth. For example, in a longitudinal study of non-Indigenous youth, Scalco et al. (2021) found support for the role of externalizing symptoms and co-occurring externalizing and internalizing symptoms in risk for alcohol use. Conversely, internalizing symptoms alone did not increase risk for alcohol use. We tried to address this limitation; however, adding externalizing symptoms to our model as a covariate resulted in non-convergence, likely due to the small number of participants and model complexity. However, this limitation, in some ways, represents the inherent trade-off between internal and external validity. Indeed, Indigenous youth represent an understudied and marginalized population. We believe that findings from the current study are critically important due to a dearth of research on this population, and we call for researchers to better include Indigenous youth going forward.

## Conclusions and Implications

We found that within individuals, higher-than-usual reported levels of drinking prospectively predicted higher levels of self-reported negative affect (depression/anxiety) than expected (accounting for their typical level of depression/anxiety) among Indigenous youth from a remote First Nations community in northern Quebec. In turn, alcohol use may precede negative affect. This temporal association may be explained by the Dual Failure Model, which suggests that adolescents who drink alcohol are more likely to experience negative consequences and in turn are at greater risk for negative affect. This risky pathway must be situated in the context of intergenerational trauma. For example, heavy consumption of alcohol was found to be more likely among Indigenous youth who had a parent or grandparent who attended an Indian Residential School (First Nations Information Governance Centre, 2018), demonstrating the devastating impacts of post-colonialism. Our findings should inform key audiences, such as community leaders and health-care providers about this issue, as well as the developers of prevention and interventions strategies. For example, similar to the work in the general health care setting, screening for negative affect (depression/anxiety) when Indigenous youth present with an alcohol use problem and vice versa should be considered best practice. However, cultural frameworks are important and majority-culture practices cannot necessarily be applied to Indigenous peoples. Beyond screening for alcohol use and negative affect, interventions and treatment programs for Indigenous youth must call on “culture as medicine” based on the history of colonization (Basset et al., 2012; Walters et al., 2020). In support of the resilience that is seen across many Indigenous communities, future research needs to continue to highlight the success and well-being of Indigenous youth and how these stories of success can help to support Indigenous youth navigate away from alcohol use, and in turn reduce depressive symptomatology.

## References

[CR1] Achenbach TM, Rescorla LA (2001). Manual for the ASEBA school-age forms & profiles.

[CR2] Achenbach TM, Rescorla LR (2007). Multicultural understanding of child and adolescent psychopathology: Implications for mental health assessment.

[CR3] Ames ME, Rawana JS, Gentile P, Morgan AS (2015). The protective role of optimism and self-esteem on depressive symptom pathways among Canadian Aboriginal youth. Journal of Youth and Adolescence.

[CR4] Anderson ER, Hope DA (2008). A review of the tripartite model for understanding the link between anxiety and depression in youth. Clinical Psychology Review.

[CR5] Andrade NN, Hishinuma ES, McDermott Jr JF, Johnson RC, Goebert DA, Makini GK, Waldron JA (2006). The National Center on Indigenous Hawaiian Behavioral Health study of prevalence of psychiatric disorders in native hawaiian adolescents. Journal of the American Academy of Child & Adolescent Psychiatry.

[CR6] Bassett D, Tsosie U, Nannauck S (2012). “Our culture is medicine”: Perspectives of Native healers on posttrauma recovery among American Indian and Alaska Native patients. The Permanente Journal.

[CR7] Beals J, Manson SM, Whitesell NR, Spicer P, Novins DK, Mitchell CM (2005). Prevalence of DSM–IV disorders and attendant help-seeking in 2 American Indian reservation populations. Archives of General Psychiatry.

[CR8] Boyd-Ball AJ, eronneau MH, Dishion TJ, Kavanagh K (2014). Monitoring and peer influences as predictors of increases in alcohol use among American Indian youth. Prevention Science.

[CR9] Brave Heart MYH (2003). The historical trauma response among natives and its relationship with substance abuse: A Lakota illustration. Journal of Psychoactive Drugs.

[CR10] Brockie TN, Dana-Sacco G, Wallen GR, Wilcox HC, Campbell JC (2015). The relationship of adverse childhood experiences to PTSD, depression, poly-drug use and suicide attempt in reservation-based native American adolescents and young adults. American Journal of Community Psychology.

[CR11] Brown RA, Dickerson DL, Klein DJ, Agniel D, Johnson CL, D’Amico EJ (2021). Identifying as American Indian/Alaska Native in urban areas: Implications for adolescent behavioral health and wellbeing. Youth & Society.

[CR12] Burack JA, Bombay A, Flores H, Stewart J, Ponizovsky V, Burack JA, Schmidt LA (2014). Developmental perspectives on the role of cultural identity in well-being: Evidence from First Nations communities in Canada. Cultural and contextual perspectives on developmental risk and well-being.

[CR13] Burack JA, Reynolds A, Landry O, Klassen G, Russo N, Fryberg S, Centifanti L, Williams D (2017). Cultural perspectives and influences on developmental psychopathology: Lessons about risk, disorder, and wellbeing from the study of the Indigenous Peoples of North America. The Wiley handbook of developmental psychopathology.

[CR14] Capaldi DM (1992). Co-occurrence of conduct problems and depressive symptoms in early adolescent boys: II. A 2-year follow-up at Grade 8. Development and Psychopathology.

[CR15] Capaldi DM, Patterson GR (1991). Relation of parental transitions to boys’ adjustment problems: I. A linear hypothesis: II. Mothers at risk for transitions and unskilled parenting. Developmental Psychology.

[CR16] Chartrand V (2019). Unsettled times: Indigenous incarceration and the links between colonialism and the penitentiary in Canada. Canadian Journal of Criminology and Criminal Justice.

[CR17] Chase, J. A. (2012). *Native American elders’ perceptions of the boarding school experience on Native American parenting: An exploratory study*. (Accession no. 390) [Doctoral Dissertation, Smith College, Northampton, Massachusetts]. Smith Scholar Works.

[CR18] Cheadle JE, Whitbeck LB (2011). Alcohol use trajectories and problem drinking over the course of adolescence: A study of north American indigennous youth and their caretakers. Journal of Health and Social Behavior.

[CR19] Chen HJ, Balan S, Price RK (2012). Association of contextual factors with drug use and binge drinking among White, native American, and mixed-race adolescents in the general population. Journal of Youth and Adolescence.

[CR20] Chorpita BF (2002). The tripartite model and dimensions of anxiety and depression: An examination of structure in a large school sample. Journal of Abnormal Child Psychology.

[CR21] Clark LA, Watson D (1991). Tripartite model of anxiety and depression: Psychometric evidence and taxonomic implications. Journal of Abnormal Psychology.

[CR22] Colder CR, Frndak S, Lengua LJ, Read JP, Hawk LW, Wieczorek WF (2018). Internalizing and externalizing problem behavior: A test of a latent variable interaction predicting a two-part growth model of adolescent substance use. Journal of Abnormal Child Psychology.

[CR23] Colder CR, Scalco M, Trucco EM, Read JP, Lengua LJ, Wieczorek WF, Hawk LW (2013). Prospective associations of internalizing and externalizing problems and their co-occurrence with early adolescent substance use. Journal of Abnormal Child Psychology.

[CR24] Cooper ML, Frone MR, Russell M, Mudar P (1995). Drinking to regulate positive and negative emotions: A motivational model of alcohol use. Journal of Personality and Social Psychology.

[CR25] Cunningham JK, Solomon TA, Muramoto ML (2016). Alcohol use among native americans compared to whites: Examining the veracity of the ‘Native American elevated alcohol consumption’ belief. Drug and Alcohol Dependence.

[CR26] Curran P, Howard A, Bainter S, Lane S, McGinley J (2014). The separation of between-person and within-person components of individual change over time: A latent curve model with structured residuals. Journal of Consulting and Clinical Psychology.

[CR27] Dishion TJ, Medici Skaggs N (2000). An ecological analysis of monthly "bursts" in early adolescent substance use. Applied Developmental Science.

[CR28] Dyer ML, Easey KE, Heron J, Hickman M, Munafò MR (2019). Associations of child and adolescent anxiety with later alcohol use and disorders: A systematic review and meta-analysis of prospective cohort studies. Addiction.

[CR29] Elliott DS, Huizinga D, Menard S (1989). Multiple problem youth: Delinquency, drugs, and mental health problems.

[CR30] Fergusson DM, Boden JM, Horwood LJ (2009). Tests of causal links between alcohol abuse or dependence and major depression. Archives of General Psychiatry.

[CR31] First Nations Information Governance Centre. (2018). *National report of the First Nations Regional Health Survey Phase 3: Volume two*. Retrived from: https://fnigc.ca/wp-content/uploads/2020/09/713c8fd606a8eeb021debc927332938d_FNIGC-RHS-Phase-III-Report1-FINAL-VERSION-Dec.2018.pdf

[CR32] Fite PJ, Colder CR, O'Connor RM (2006). Childhood behavior problems and peer selection and socialization: Risk for adolescent alcohol use. Addictive Behaviors.

[CR33] Goldstein SC, Schick MR, Nalven T, Spillane NS (2021). The role of valuing cultural activities in the association between alcohol expectancies and alcohol use among First Nation adolescents. Journal of Studies on Alcohol and Drugs.

[CR34] Gone JP (2021). The (post)colonial predicament in community mental health services for American indians: Explorations in alter-native psy-ence. The American Psychologist.

[CR35] Greenfield BL, Sittner KJ, Forbes MK, Walls ML, Whitbeck LB (2017). Conduct disorder and alcohol use disorder trajectories, predictors, and outcomes for indigenous youth. Journal of the American Academy of Child & Adolescent Psychiatry.

[CR36] Hautala D, Sittner K, Walls M (2019). Onset, comorbidity, and predictors of nicotine, alcohol, and marijuana use disorders among north American indigenous adolescents. Journal of Abnormal Child Psychology.

[CR37] HeavyRunner-Rioux AR, Hollist DR (2010). Community, family, and peer influences on alcohol, marijuana, and illicit drug use among a sample of native American youth: An analysis of predictive factors. Journal of Ethnicity in Substance Abuse.

[CR38] Hop Wo NK, Anderson KK, Wylie L, MacDougall A (2020). The prevalence of distress, depression, anxiety, and substance use issues among indigenous post-secondary students in Canada. Transcultural Psychiatry.

[CR39] Hu LT, Bentler PM (1999). Cutoff criteria for fit indexes in covariance structure analysis: Conventional criteria versus new alternatives. Structural Equation Modeling.

[CR40] Indian Health Service. (2018). *Report on behavioral health: 2018 edition*. Retrieved from https://www.ihs.gov/sites/dps/themes/responsive2017/display_objects/documents/IHS2018BehavioralHealth.pdf

[CR41] Kenney MK, Singh GK (2016). Adverse childhood experiences among American Indian/Alaska native children: The 2011–2012 National Survey of children’s Health. Scientifica.

[CR42] Khantzian EJ (1985). The self-medication hypothesis of addictive disorders: Focus on heroin and cocaine dependence. The American Journal of Psychiatry.

[CR43] Khantzian EJ (1997). The self-medication hypothesis of substance use disorders: A reconsideration and recent applications. Harvard Review of Psychiatry.

[CR44] Kirmayer LJ, Dandeneau S, Marshall E, Phillips MK, Williamson KJ (2011). Rethinking resilience from indigenous perspectives. The Canadian Journal of Psychiatry.

[CR45] Kline RB (2010). Principles and practice of structural equation modeling.

[CR46] Kobus K (2003). Peers and adolescent smoking. Addiction (Abingdon, England).

[CR47] Kuntsche E, Knibbe R, Gmel G, Engels R (2005). Why do young people drink? A review of drinking motives. Clinical Psychology Review.

[CR48] Landen M, Roeber J, Naimi T, Nielsen L, Sewell M (2014). Alcohol-attributable mortality among American indians and Alaska Natives in the United States, 1999–2009. American Journal of Public Health.

[CR49] Lavell-Harvard, D. M., & Brant, J. (Eds.). (2016). *Forever loved: Exposing the hidden crisis of missing and murdered indigenous women and girls in Canada*. Ontario, Canada: Demeter Press.

[CR50] Leve LD, Kim HK, Pears KC (2005). Childhood temperament and family environment as predictors of internalizing and externalizing trajectories from ages 5 to 17. Journal of Abnormal Child Psychology.

[CR51] Louie DW, Gereluk D (2021). The insufficiency of high school completion rates to redress educational inequities among indigenous students. Philosophical Inquiry in Education.

[CR52] Ma J (2021). The intersection and parallels of aboriginal peoples’ and racialized migrants’ experiences of colonialism and child welfare in Canada. International Social Work.

[CR53] Marmorstein NR (2009). Longitudinal associations between alcohol problems and depressive symptoms: Early adolescence through early adulthood. Alcoholism, Clinical and Experimental Research.

[CR54] Marsh HW, Wen Z, Hau KT (2004). Structural equation models of latent interactions: evaluation of alternative estimation strategies and indicator construction. Psychological Methods.

[CR55] Martinez MM, Armenta BE (2020). Trajectories of depressive symptoms among north American indigenous adolescents: Considering predictors and outcomes. Child Development.

[CR56] Mason WA, Hitchings JE, Spoth RL (2008). The interaction of conduct problems and depressed mood in relation to adolescent substance involvement and peer substance use. Drug and Alcohol Dependence.

[CR57] McCabe CJ, Brumback T, Brown SA, Meruelo AD (2023). Assessing cross-lagged associations between depression, anxiety, and binge drinking in the National Consortium on Alcohol and Neurodevelopment in Adolescence (NCANDA) study. Drug and Alcohol Dependence.

[CR58] McCarty C, Wymbs B, King K, Mason WA, Stoep A, McCauley E (2012). Developmental consistency in associations between depressive symptoms and alcohol use in early adolescence. Journal of Studies on Alcohol and Drugs.

[CR59] McCarty CA, Wymbs BT, Mason WA, King KM, McCauley E, Baer J (2013). Early adolescent growth in depression and conduct problem symptoms as predictors of later substance use impairment. Journal of Abnormal Child Psychology.

[CR60] McLaughlin KA, King K (2015). Developmental trajectories of anxiety and depression in early adolescence. Journal of Abnormal Child Psychology.

[CR61] Muthén, L. K., & Muthén, B. O. (1998–2018). *Mplus User’s Guide* (8th ed.). Los Angeles, CA: Muthén and Muthén.

[CR62] O’Neil KA, Conner BT, Kendall PC (2011). Internalizing disorders and substance use disorders in youth: Comorbidity, risk, temporal order, and implications for intervention. Clinical Psychology Review.

[CR63] Oetting ER, Beauvais F (1991). Orthogonal cultural identification theory: The cultural identification of minority adolescents. International Journal of the Addictions.

[CR64] Paige KJ, Meisel SN, Colder CR (2021). An examination of reciprocal associations between substance use and effortful control across adolescence using a bifactor model of externalizing symptoms. Development and Psychopathology.

[CR65] Razack SH (2016). Sexualized violence and colonialism: Reflections on the inquiry into missing and murdered indigenous women. Canadian Journal of Women and the Law.

[CR66] Rice F, Riglin L, Thapar AK, Heron J, Anney R, O’Donovan MC, Thapar A (2019). Characterizing developmental trajectories and the role of neuropsychiatric genetic risk variants in early-onset depression. JAMA Psychiatry.

[CR67] Rieckmann T, McCarty D, Kovas A, Spicer P, Bray J, Gilbert S, Mercer J (2012). American indians with substance use disorders: Treatment needs and comorbid conditions. The American Journal of Drug and Alcohol Abuse.

[CR68] Ross A, Dion J, Cantinotti M, Collin-V zina D, Paquette L (2015). Impact of residential schooling and of child abuse on substance use problem in Indigenous peoples. Addictive Behaviors.

[CR69] Salk RH, Hyde JS, Abramson LY (2017). Gender differences in depression in representative national samples: Meta-analyses of diagnoses and symptoms. Psychological Bulletin.

[CR70] Scalco M, Colder C, Read J, Lengua L, Wieczorek W, Hawk L (2021). Testing alternative cascades from internalizing and externalizing symptoms to adolescent alcohol use and alcohol use disorder through co-occurring symptoms and peer delinquency. Development and Psychopathology.

[CR71] Schick MR, Nalven T, Thomas ED, Weiss NH, Spillane NS (2022). Depression and alcohol use in American Indian adolescents: The influence of family factors. Alcoholism: Clinical & Experimental Research.

[CR72] Schleider JL, Ye F, Wang F, Hipwell AE, Chung T, Sartor CE (2019). Longitudinal reciprocal associations between anxiety, Depression, and Alcohol Use in adolescent girls. Alcoholism Clinical and Experimental Research.

[CR73] Scott, B. G., Sunchild, L., Small, C., & McCullen, J. R. (2022). Anxiety and depression in Northern Plains American Indian Youth: Evidence for resilience and risk. *Journal of Clinical Child & Adolescent Psychology,* 1–13. 10.1080/15374416.2022.2127101 10.1080/15374416.2022.2127101 PMC10079783 36206519

[CR74] Shore L, Toumbourou JW, Lewis AJ, Kremer P (2018). Longitudinal trajectories of child and adolescent depressive symptoms and their predictors – A systematic review and meta-analysis. Child and Adolescent Mental Health.

[CR75] Singh GK, Daus GP, Allender M, Ramey CT, Martin EK, Perry C, Vedamuthu IP (2017). Social determinants of health in the United States: Addressing major health inequality trends for the nation, 1935–2016. International Journal of MCH and AIDS.

[CR76] Spillane NS, Kirk-Provencher KT, Schick MR, Nalven T, Goldstein SC, Kahler CW (2020). Identifying competing life reinforcers to substance use in First Nation adolescents. Substance Use & Misuse.

[CR77] Stanley LR, Harness SD, Swaim RC, Beauvais F (2014). Rates of substance use of American Indian students in 8th, 10th, and 12th grades living on or near reservations: Update, 2009–2012. Public Health Reports.

[CR78] Stapinski LA, Araya R, Heron J, Montgomery AA, Stallard P (2015). Peer victimization during adolescence: Concurrent and prospective impact on symptoms of depression and anxiety. Anxiety Stress and Coping.

[CR79] Statistics Canada. (2021). *Indigenous population in Canada–Projections to 2041*. Retrieved from: https://www150.statcan.gc.ca/n1/pub/11-627-m/11-627-m2021066-eng.htm

[CR80] Stewart SH, Sherry SB, Comeau MN, Mushquash CJ, Collins P, Van Wilgenburg H (2011). Hopelessness and excessive drinking among aboriginal adolescents: The mediating roles of depressive symptoms and drinking to cope. Depression Research and Treatment.

[CR81] Substance Abuse and Mental Health Services Administration. (2019). *Key substance use and mental health indicators in the United States: Results from the 2018 National Survey on Drug Use and Health* (HHS Publication No. PEP19-5068, NSDUH Series H-54). Rockville, MD: Center for Behavioral Health Statistics and Quality, Substance Abuse and Mental Health Services Administration. Retrieved from https://www.samhsa.gov/data/sites/default/files/cbhsq-reports/NSDUHNationalFindingsReport2018/NSDUHNationalFindingsReport2018.pdf

[CR82] Szlemko WJ, Wood JW, Thurman PJ (2006). Native americans and alcohol: Past, present, and future. Journal of General Psychology.

[CR83] Thira D (2014). Aboriginal youth suicide prevention: A post-colonial community-based approach. International Journal of Child, Youth and Family Studies.

[CR84] Thrane LE, Whitbeck LB, Hoyt DR, Shelley MC (2004). Comparing three measures of depressive symptomology among American Indian adolescents. American Indian & Alaska Native Mental Health Research.

[CR85] Tjepkema M, Bushnik T, Bougie E (2019). Life expectancy of First Nations, Metis, and Inuit household populations in Canada. Statistics Canada: Health Reports.

[CR86] Walls M, Sittner KJ, Whitbeck LB, Herman K, Gonzalez M, Elm JHL, Hoyt DR (2021). Prevalence of mental disorders from adolescence through early adulthood in American Indian and first nations communities. International Journal of Mental Health and Addiction.

[CR87] Walters KL, Johnson-Jennings M, Stroud S, Rasmus S, Charles B, John S, Boulafentis J (2020). Growing from our roots: Strategies for developing culturally grounded health promotion interventions in American Indian, Alaska Native, and native hawaiian communities. Prevention Science.

[CR88] Warne D, Lajimodiere D (2015). American Indian health disparities: Psychosocial influences. Social & Personality Psychology Compass.

[CR89] Warne D, Dulacki K, Spurlock M, Meath T, Davis MM, Wright B, McConnell KJ (2017). Adverse childhood experiences (ACE) among American indians in South Dakota and associations with mental health conditions, alcohol use, and smoking. Journal of Health Care for the Poor and Underserved.

[CR90] Watson D (2005). Rethinking the mood and anxiety disorders: A quantitative hierarchical model for DSM-V. Journal of Abnormal Psychology.

[CR91] Whitbeck LB, Adams GW, Hoyt DR, Chen X (2004). Conceptualizing and measuring historical trauma among American Indian people. American Journal of Community Psychology.

[CR92] Whitbeck LB, Hartshorn S, Crawford KJ, Walls DM, Gentzler ML, Hoyt DR (2014). Mental and substance use disorders from early adolescence to young adulthood among indigenous young people: Final diagnostic results from an 8-year panel study. Social Psychiatry and Psychiatric Epidemiology.

[CR93] White AM (2020). Gender differences in the epidemiology of alcohol use and related harms in the United States. Alcohol Research: Current Reviews.

[CR94] Wiechelt SA, Gryczynski J, Johnson JL, Caldwell D (2012). Historical trauma among urban American indians: Impact on substance abuse and family cohesion. Journal of Loss and Trauma.

